# Data on enterobacteria activity on biofilm formation at surface mango fruit (*Mangifera indica* L.) cv Ataulfo

**DOI:** 10.1016/j.dib.2016.10.014

**Published:** 2016-10-26

**Authors:** Juan A. Ragazzo-Sánchez, Ramsés R. González-Estrada, Martha A. Santana-Martínez, María del Carmen Wacher-Rodarte, Carlos A. Eslava-Campos, Montserrat Calderón-Santoyo

**Affiliations:** a Laboratorio Integral de Investigación en Alimentos. Instituto Tecnológico de Tepic, Av. Tecnológico No. 2595, Tepic, Nay. C.P. 63175, México; b Departamento de Salud Pública, Facultad de Medicina, Universidad Nacional Autónoma de México, Mexico City, Mexico; c Lab 324, Conjunto E, Departamento de Alimentos y Biotecnología, Facultad de Química, Universidad Nacional Autónoma de México, Mexico City, Mexico

**Keywords:** Biofilms, Biotic surfaces, Mango

## Abstract

Abiotic factors influenced the capacity of the strains to form biofilms. Classification of the adhesion type is related with the optical density measured on the biofilm formation of tested strains. The relationship between the biofilm formation in real values with theoretical values of the strains was used to determine the mechanism involved during mixed cultures.

**Specifications Table**

**Table:** 

Subject area	*Microbiology*
More specific subject area	*Postharvest*
Type of data	*text file, table*
How data was acquired	*Spectrophotometer (Cary Model 50)*
Data format	*Analyzed*
Experimental factors	*Effect of pH, temperature, nutrients addition and microbial interactions on formation of biofilms on mango surface.*
Experimental features	One-way analysis of variance (ANOVA)
Data source location	*Tepic, Nayarit; México* (21º 30’ 0” N, 104º 54’ 0” W).
Data accessibility	*Data is with this article*

**Value of the data**

The data provide useful information for producers and consumers concerning biofilms formation on mango fruits.
The presence of these Enterobacteria reminds the importance of the application of good agricultural practices.
Effect of microbial interactions on biofilm formation are important for the industry considering the impact to human health.

## 1 Data

The optical density of cristal violeta absorbed by Enterobacteria involved in the biofilm formation at surface fruit, by individual and mixed strains and also the mechanism of interaction between strains during biofilms formation.

## 2 Experimental design, materials and methods

All experiments were conducted at least in 3 independent times in duplicates. The data were analyzed with the statistical software SAS v. 9.0. Statistical significance was determined by Tukey test using one-way analysis of variance (ANOVA). Fruits were harvested and selected without damage. Enterobacteria were previously isolated from mango surface. The type of mechanism between strains was assessed comparing the amount of biofilm formation in absorbance units [1], [2]. Influence of abiotic factors as temperature, pH and nutrients were considered for the evaluation (see Supplementary Table 1). As biotic factor the interactions between strains was assessed. The determination of the mechanism between strains, a comparison of the amount of biofilm of the theoretical value and mixed the real value were performed (see Supplementary Table 2) (Table 1, Table 2).

## Figures and Tables

**Table 1:** Influence of abiotic factors in biofilm formation on surface mango fruit.

Optical density at 590 nm							
Strain	Temperature (°C)			pH		Nutrients	
	12	25	33	5	7	With	Without
*E*. *coli*	0.310±0.034^b^	1.232±0.230^a^	1.165±0.056^a^	0.825±0.150^a^	0.98±0.125^a^	0.691±0.150^a^	1.114±0.320^a^
*P*. *aeruginosa*	0.465±0.023^b^	1.068±0.070^a^	0.614±0.150^ab^	0.514±0.140^b^	0.918±0.195^a^	0.498±0.050^b^	0.934±0.185^a^
*K*. *pneumoniae*	0.703±0.014^a^	0.580±0.030^a^	0.444±0.025^a^	0.328±0.050^b^	0.824±0.035^a^	0.172±0.025^b^	0.980±0.150^a^
*E*. *aerogenes*	0.448±0.140^a^	0.367±0.080^a^	0.585±0.020^a^	0.622±0.0150^a^	0.311±0.0122^a^	0.477±0.050^a^	0.456±0.070^a^
*Salmonella* spp.	0.114±0.005^b^	0.773±0.050^a^	0.569±0.038^a^	0.377±0.004^a^	0.593±0.080^a^	0.471±0.009^a^	0.499±0.120^a^

Mean followed by the same letter in a row for each factor is not significantly different (Tukey, *P* > 0.05).

**Table 2:** Mechanism involved of mixed-culture strains in biofilm production on mango surface.

Mixed strains	Biofilm production	Mechanism
	Theoretical/Real	
Ec–S	1.760±0.012 < 9.341±1.061	Protocooperation
Ea–Ec	1.557±0.005 < 9.066±1.043	Protocooperation
Ea–S	0.741±0.430 < 8.901±1.250	Protocooperation
Kp–Ea	1.099±0.003 < 5.291±1.050	Protocooperation
Pa–S	1.540±0.020 < 4.079±0.650	Protocooperation
Pa–Ec	2.356±0.001 ≥ 2.334±0.450	Neutralism
Ea–Pa	1.337±0.520 < 2.200±0.750	Protocooperation
Kp–Pa	1.898±0.030 < 2.011±0.500	Protocooperation
Kp–Ec	2.119±0.500 > 0.685±0.300	Competition
Kp–S	1.302±0.432 > 0.033±0.001	Competition
Kp–Pa–S	1.580±0.033 < 9.445±1.250	Protocooperation
Ea–Pa–Ec	1.571±0.021 < 9.426±0.950	Protocooperation
Ea–Pa–S	1.206±0.051 < 9.086±0.350	Protocooperation
Kp–Ea–Ec	1.523±0.049 < 6.613±1.150	Protocooperation
Pa–Ec–S	1.885±0.00 < 5.983±0.950	Protocooperation
Kp–a–Pa	1.445±0.022 < 5.389±1.045	Protocooperation
Kp–Ec–S	1.727±0.011 < 5.165±0.450	Protocooperation
Kp–Ea–S	1.047±0.035 < 2.230±0.050	Protocooperation
Ea–Ec–S	1.353±0.025 < 1.713±0.030	Protocooperation
Kp–Pa–Ec	2.124±0.056 > 1.430±0.050	Competition

Average OD_590_, optical density at 590 nm; STD, Standard deviation. Three repetitions were performed for each mix. Ec= *E. coli* 06:H36, S= *Salmonella* spp., Ea= *E. aerogenes* , Kp= *K. pneumoniae*; Pa= *P. aeuroginosa*.
