# Supplementary material for: Data on enterobacteria activity on biofilm formation at surface mango fruit (Mangifera indica L.) cv Ataulfo
Source: Data Brief. 2016 Oct 26;9:746–8. doi: 10.1016/j.dib.2016.10.014 (PMC5096593; doi:10.1016/j.dib.2016.10.014)
Supplement: Supplementary file 2 — Supplementary material [file mmc2.docx]

| Optical density at 590 nm |
| --- |

| Strain |  | Temperature (°C) | | | pH | | Nutrients | |
| --- | --- | --- | --- | --- | --- | --- | --- | --- |
|  |  | 12 | 25 | 33 | 5 | 7 | With | Without |

| E. *coli* | 0.310±0.034^b^ | 1.232±0.230^a^ | 1.165±0.056^a^ | 0.825±0.150^a^ | 0.98±0.125^a^ | 0.691±0.150^a^ | 1.114±0.320^a^ |
| --- | --- | --- | --- | --- | --- | --- | --- |
| P. *aeruginosa* | 0.465±0.023^b^ | 1.068±0.070^a^ | 0.614±0.150^ab^ | 0.514±0.140^b^ | 0.918±0.195^a^ | 0.498±0.050^b^ | 0.934±0.185^a^ |
| K. *pneumoniae* | 0.703±0.014^a^ | 0.580±0.030^a^ | 0.444±0.025^a^ | 0.328±0.050^b^ | 0.824±0.035^a^ | 0.172±0.025^b^ | 0.980±0.150^a^ |
| E. *aerogenes* | 0.448±0.140^a^ | 0.367±0.080^a^ | 0.585±0.020^a^ | 0.622±0.0150^a^ | 0.311±0.0122^a^ | 0.477±0.050^a^ | 0.456±0.070^a^ |
| *Salmonella* spp. | 0.114±0.005^b^ | 0.773±0.050^a^ | 0.569±0.038^a^ | 0.377±0.004^a^ | 0.593±0.080^a^ | 0.471±0.009^a^ | 0.499±0.120^a^ |

Mean followed by the same letter in a row for each factor is not significantly different (Tukey, 𝑃 > 0.05).
