# Supplementary material for: Data on enterobacteria activity on biofilm formation at surface mango fruit (Mangifera indica L.) cv Ataulfo
Source: Data Brief. 2016 Oct 26;9:746–8. doi: 10.1016/j.dib.2016.10.014 (PMC5096593; doi:10.1016/j.dib.2016.10.014)
Supplement: Supplementary file 3 — Supplementary material [file mmc3.docx]

| Mixed strains | Biofilm production  Theoretical/Real | Mechanism |
| --- | --- | --- |
| Ec-S | 1.760±0.012 < 9.341±1.061 | Protocooperation |
| Ea-Ec | 1.557±0.005 < 9.066±1.043 | Protocooperation |
| Ea-S | 0.741±0.430 < 8.901±1.250 | Protocooperation |
| Kp-Ea | 1.099±0.003 < 5.291±1.050 | Protocooperation |
| Pa-S | 1.540±0.020 < 4.079±0.650 | Protocooperation |
| Pa-Ec | 2.356±0.001 ≥ 2.334±0.450 | Neutralism |
| Ea-Pa | 1.337±0.520 < 2.200±0.750 | Protocooperation |
| Kp-Pa | 1.898±0.030 < 2.011±0.500 | Protocooperation |
| Kp-Ec | 2.119±0.500 > 0.685±0.300 | Competition |
| Kp-S | 1.302±0.432 > 0.033±0.001 | Competition |
| Kp-Pa-S | 1.580±0.033 < 9.445±1.250 | Protocooperation |
| Ea-Pa-Ec | 1.571±0.021 < 9.426±0.950 | Protocooperation |
| Ea-Pa-S | 1.206±0.051 < 9.086±0.350 | Protocooperation |
| Kp-Ea-Ec | 1.523±0.049 < 6.613±1.150 | Protocooperation |
| Pa-Ec-S | 1.885±0.00 < 5.983±0.950 | Protocooperation |
| Kp-Ea-Pa | 1.445±0.022 < 5.389±1.045 | Protocooperation |
| Kp-Ec-S | 1.727±0.011 < 5.165±0.450 | Protocooperation |
| Kp-Ea-S | 1.047±0.035 < 2.230±0.050 | Protocooperation |
| Ea-Ec-S | 1.353±0.025 < 1.713±0.030 | Protocooperation |
| Kp-Pa-Ec | 2.124±0.056 > 1.430±0.050 | Competition |

Average OD_590_, optical density at 590 nm; STD, Standard deviation. Three repetitions were performed for each mix. Ec= *E. coli* 06:H36, S= *Salmonella* spp., Ea= *E. aerogenes* , Kp= *K. pneumoniae*; Pa= *P. aeuroginosa*.
